# The Promher Study: An Observational Italian Study on Adjuvant Therapy for HER2-Positive, pT1a-b pN0 Breast Cancer

**DOI:** 10.1371/journal.pone.0136731

**Published:** 2015-09-04

**Authors:** Stefania Gori, Alessandro Inno, Elena Fiorio, Jennifer Foglietta, Antonella Ferro, Marcella Gulisano, Graziella Pinotti, Marta Gubiotti, Maria Giovanna Cavazzini, Monica Turazza, Simona Duranti, Valeria De Simone, Laura Iezzi, Giancarlo Bisagni, Simon Spazzapan, Luigi Cavanna, Chiara Saggia, Emilio Bria, Elisabetta Cretella, Patrizia Vici, Daniele Santini, Alessandra Fabi, Ornella Garrone, Antonio Frassoldati, Laura Amaducci, Silvana Saracchini, Lucia Evangelisti, Sandro Barni, Teresa Gamucci, Lucia Mentuccia, Lucio Laudadio, Alessandra Zoboli, Fabiana Marchetti, Giuseppe Bogina, Gianluigi Lunardi, Luca Boni

**Affiliations:** 1 Department of Oncology, Ospedale Sacro Cuore don Calabria, Negrar–Verona, Italy; 2 Unit of Oncology, Ospedale Civile Maggiore, Verona, Italy; 3 Medical Oncology, Azienda Ospedaliera di Perugia, Perugia, Italy; 4 Medical Oncology, Ospedale Santa Chiara, Trento, Italy; 5 Medical Oncology, Ospedale S. Bortolo, Vicenza, Italy; 6 Department of Oncology, Ospedale di Circolo and University of Insubria, Varese, Italy; 7 Medical Oncology, Ospedale Civile di Macerata, Macerata, Italy; 8 Unit of Hematology and Oncology, AO Carlo Poma, Mantova, Italy; 9 Medical Oncology, Ospedale San Donato, Arezzo, Italy; 10 Medical Oncology, Ospedale SS Annunziata, Chieti, Italy; 11 Medical Oncology, Breast Unit, Arcispedale S. Maria Nuova, Reggio Emilia, Italy; 12 Medical Oncology, C.R.O.–I.R.C.C.S., Aviano–Pordenone, Italy; 13 Department of Oncology and Hematology, Ospedale Guglielmo da Saliceto, Piacenza, Italy; 14 Medical Oncology, Ospedale Maggiore della Carità, Novara, Italy; 15 Department of Medicine, Medical Oncology, University of Verona, Azienda Ospedaliera Universitaria Integrata, Verona, Italy; 16 Medical Oncology, Azienda Sanitaria dell’Alto Adige, Bolzano, Italy; 17 Division of Medical Oncology B, Regina Elena National Cancer Institute, Roma, Italy; 18 Department of Medical Oncology, Campus Bio-Medico University, Roma, Italy; 19 Division of Medical Oncology A, Regina Elena National Cancer Institute, Roma, Italy; 20 Division of Medical Oncology, Azienda Ospedaliera S. Croce e Carle, Cuneo, Italy; 21 Medical Oncology, University Hospital, Ferrara, Italy; 22 Medical Oncology, Ospedale per gli Infermi, Faenza–Ravenna, Italy; 23 Medical Oncology, Ospedale Santa Maria degli Angeli, Pordenone, Italy; 24 Medical Oncology, ASL CN1, Saluzzo–Cuneo, Italy; 25 Medical Oncology, Azienda Ospedaliera Treviglio, Treviglio–Bergamo, Italy; 26 Medical Oncology Unit, ASL Frosinone, Frosinone, Italy; 27 Department of Oncology, Ospedale Floraspe Renzetti, Lanciano–Chieti, Italy; 28 Medical Oncology, Ospedale San Sebastiano, Correggio–Reggio Emilia, Italy; 29 Pathology, Ospedale Sacro Cuore don Calabria, Negrar–Verona, Italy; 30 Azienda Ospedaliero–Universitaria Careggi, Firenze, Italy; University of Torino, ITALY

## Abstract

**Background:**

The management of pT1a-b pN0 HER2-positive breast cancer is controversial and no data about the efficacy of trastuzumab in this setting are available from randomized clinical trials. The aims of this retrospective study were to assess how patients are managed in clinical practice in Italy, which clinical or biological characteristics influenced the choice of adjuvant systemic therapy and the outcome of patients.

**Methods:**

Data of consecutive patients who underwent surgery from January 2007 to December 2012 for HER2-positive, pT1a-b pN0 M0 breast cancer were retrospectively collected from 28 Italian centres. Analysis of contingency tables and multivariate generalized logit models were used to investigate the association between the baseline clinical and biological features and the treatment strategy adopted.

**Results:**

Among 303 enrolled patients, 204 received adjuvant systemic therapy with trastuzumab, 65 adjuvant systemic therapy without trastuzumab and 34 did not receive adjuvant systemic therapy. At the multivariate analysis age, tumor size, proliferation index and hormone receptor status were significantly associated with the treatment choice. Five-year disease-free survival (DFS) probability was 95%, 94.3% and 69.6% for patients treated with adjuvant systemic therapy and trastuzumab, with adjuvant systemic therapy without trastuzumab and for patients who did not receive adjuvant systemic therapy, respectively (p<0.001).

**Conclusions:**

The majority of patients (66%) with pT1a-b pN0 HER2-positive breast cancer enrolled in this retrospective study received adjuvant systemic therapy with trastuzumab, whereas only 11% patients did not receive any adjuvant systemic therapy. The choice of treatment type seems to be mainly influenced by tumor size, proliferation index, hormone receptor status and age. The 5-year DFS probability was significantly higher for patients receiving adjuvant systemic therapy with trastuzumab compared with patients not receiving adjuvant systemic therapy or receiving adjuvant systemic therapy without trastuzumab.

## Introduction

Mainly due to increasing breast cancer awareness and widespread mammographic screening, a growing number of women have been diagnosed over the last 15 years with pT1a-b pN0 breast cancer (0.1–0.5 cm, T1a; or >0.5–1.0 cm, T1b [1]). The incidence of such small tumors is expected to further increase in the next future [2]. In general, pT1a-b pN0 breast cancer has an excellent prognosis, with a cancer-related mortality rate at 10 years of less than 5% [3].

Human Epidermal Growth Factor Receptor-2 (HER2) positive status, defined as HER2 protein overexpression (immunohistochemistry 3+) and/or *HER2* gene amplification, is observed in about 15–25% of breast carcinomas overall [4], and less frequently in ≤ 1 cm breast cancer (10%) [5,6].

HER2-positive status is associated with worse prognosis, even for small tumors. However, the magnitude of the impact of HER2 status on the prognosis of pT1a-b pN0 breast cancer is highly variable in different studies. In the MD Anderson series of 98 patients with HER2-positive pT1a-b pN0 breast cancer, 5-year recurrence-free and distant recurrence-free survival rates were 77.1% and 86.4%, respectively [6]. A 9-year distant disease-free survival rate of 67% was reported by Joensuu et al for patients with G2–3 HER2-positive pT1a-b breast cancer [7]. Furthermore, a pooled analysis of 764 patients with pT1a-b pN0 breast cancer showed that HER2-positive status was associated with a significant detrimental effect on relapse-free and distant relapse-free survival (hazard ratio 4.68 and 5.6, respectively) and on breast cancer specific survival (hazard ratio 2.61) [8]. However, other studies reported relatively good outcomes for patients with HER2-positive small breast cancer, especially for those with pT1a and hormone receptor positive tumors. In fact, data from the Kaiser Permanente Clinical Care Program of Northern California showed a 5-year relapse-free interval rates of 97.4% for pT1a and of 90.9% for pT1b HER2-positive tumors [9]. A prospective cohort study within the National Comprehensive Cancer Network Database reported 5-year invasive disease relapse-free interval rates of 86% for patients with both pT1a and pT1b hormone receptor positive / HER2-positive tumors, and of 84% and 64% for patients with hormone receptor negative / HER2-positive pT1a and pT1b tumors, respectively [10].

Several randomized phase III clinical trials have clearly demonstrated that trastuzumab, a monoclonal antibody directed against the extracellular domain of HER2, meaningfully improves distant-free and overall survival when added to standard adjuvant chemotherapy for HER2-positive early breast cancer [11–20].

Population with pT1a-b pN0 breast cancer was not adequately represented in randomized trials investigating the efficacy of adjuvant trastuzumab, since most of the studies included only node positive or high risk node negative breast cancers with primary tumor size ≥ 2 cm.

Some retrospective studies [21–23] and a meta-analysis of retrospective trials [24] have suggested a benefit from adjuvant trastuzumab for patients with HER2-positive pT1a-b pN0 breast cancer. However, since no data from prospective randomized clinical trials are available, the optimal management of these tumors is still controversial.

We designed the present retrospective study to identify the clinicopathological features influencing Italian medical oncologists in the choice of the adjuvant systemic therapy with or without trastuzumab for patients with HER2-positive, pT1a-b pN0 breast cancer in clinical practice. A secondary objective of the study was to investigate any difference in terms of outcome according to the type of adjuvant systemic therapy.

## Methods

### Ethics statement

The study protocol was approved by the ethics committee of Verona and Rovigo area. Being a retrospective analysis of clinical outcomes, no specific written informed consent was required. Patients records were anonymized and de-identified prior to analysis.

### Patients and methods

Data of consecutive patients who underwent surgery from January 2007 to December 2012 for breast cancer were collected through a specifically developed web-based case report form system from 28 Italian sites, including both academic and non-academic oncology units representative of the national clinical practice. Patients were eligible if at the pathology examination of surgical specimen they had ≤ 1 cm in size breast cancer, negative nodal status assessed with sentinel lymph node biopsy (SLB) or axillary lymph nodes dissection (ALD), HER2-positive (immunohistochemistry 3+ and/or FISH amplified) disease. Main exclusion criteria were any neoadjuvant treatment, *in situ* breast carcinoma or micro-invasive carcinoma (i.e. ≤ 1 mm of invasive tumour). According to the American Joint Committee on Cancer, tumor size was defined as follows: 0.1–0.5 cm, pT1a and >0.5–1.0 cm, pT1b [1]. Tumors were considered hormone receptor positive if estrogen and/or progesterone receptor were positive in ≥ 1% of cancer cells at immunohistochemistry. A Ki67 cut-off value of ≥ 14% was considered to define tumors with high proliferation rate.

Three groups of treatment were defined according to the adjuvant systemic therapy, as follows: no adjuvant systemic therapy, if the patients did not received any medical treatment in the adjuvant setting; adjuvant systemic therapy without trastuzumab, if the patients received chemotherapy and/or endocrine therapy in the adjuvant setting; adjuvant systemic therapy with trastuzumab if the patients received chemotherapy and/or endocrine therapy plus the anti-HER2 antibody.

Disease-free survival was defined as the time from surgery for breast cancer until first local and/or distance recurrence or death for any cause if it happened before recurrence.

### Statistical analysis

At the univariate analysis, the presence of association between the baseline clinical and biological characteristics and the treatment strategy adopted was evaluted by χ^2^ test for heterogenity, Fisher exact test when appropriate, and Krukal-Wallis test, for categorical and continuous variables respectively. The multivariate analysis was performed using a multinomial logistic regression model and missing values were handled by single imputation technique. Median follow-up time and its interquartile range (IQR) were estimated according to the Kaplan–Meier inverse method. Estimates of DFS were calculated according to the Kaplan-Meier product-limit method. Comparisons of survival curves were performed with the log-rank test. P values of the paired tests were adjusted for multiple-comparison according to the Scheffè's method. The p values are 2-sided and considered statistically significant at <0.05. Data were analyzed using the statistical software SAS 9.2 (SAS Corporation, Cary, NC).

## Results

### Characteristics of patients and systemic adjuvant therapy

Patients and tumor characteristics are summarized in Table 1. Among 303 enrolled patients, median age at surgery was 57 years, and 66% of patients were post-menopausal. Tumor size was pT1a for 97 (32%) and pT1b for 204 (67%) patients. Data on tumor size were not available for 2 patients, but both of them had tumors less than 1 cm in size according to the inclusion criteria. Most of tumors were ductal carcinoma (95%), with moderate (G2, 46%) or high grade (G3 47%), and high proliferative index (74%).

**Table 1:** Clinicopathologic characteristics and univariate analysis.

Variable	All patients, n = 303	No AST, n = 34	AST without trastuzumab, n = 65	AST with trastuzumab, n = 204	p value (*)
**Median age, yrs (range)**	57 (31–84)	59 (35–84)	59 (36–84)	55 (31–79)	0.003
**Menopausal status:**					0.047
Pre	95 (31%)	7 (21%)	15 (23%)	73 (36%)	
Post	201 (66%)	26 (76%)	50 (77%)	125 (61%)	
Unknown	7 (2%)	1 (3%)	0 (0%)	6 (3%)	
**pT size:**					<0.001
pT1a	97 (32%)	29 (85%)	22 (34%)	46 (23%)	
pT1b	204 (67%)	5 (15%)	43 (66%)	156 (76%)	
Unknown	2 (1%)	0 (0%)	0 (0%)	2 (1%)	
**Histology:**					0.009
Ductal	288 (95%)	29 (85%)	62 (95%)	197 (97%)	
Lobular	5 (2%)	0 (0%)	1 (2%)	4 (2%)	
Other	10 (3%)	5 (15%)	2 (3%)	3 (1%)	
**Tumor grade:**					<0.001
G1	14 (5%)	2 (6%)	5 (8%)	7 (3%)	
G2	140 (46%)	8 (23%)	44 (68%)	88 (43%)	
G3	144 (47%)	22 (65%)	16 (24%)	106 (52%)	
Gx	5 (2%)	2 (6%)	0 (0%)	3 (1%)	
**Ki67:**					0.015
<14%	74 (24%)	10 (29%)	25 (38%)	39 (19%)	
≥14%	224 (74%)	24 (71%)	38 (58%)	162 (79%)	
Unknown	5 (2%)	0 (0%)	2 (3%)	3 (1%)	
**LVI:**					0.255
No	213 (70%)	26 (76%)	47 (72%)	140 (69%)	
Yes	21 (7%)	0 (0%)	5 (8%)	16 (8%)	
Unknown	69 (23%)	8 (24%)	13 (20%)	48 (23%)	
**HR status:**					<0.001
ER-/PgR –	100 (33%)	30 (88%)	3 (5%)	67 (33%)	
ER + and/or PgR +	200 (66%)	4 (12%)	61 (94%)	135 (66%)	
Unknown	3 (1%)	0 (0%)	1 (1%)	2 (1%)	

LVI: lymphovascular invasion; HR: hormone receptor; AST: adjuvant systemic therapy; ER: estrogen receptor; PgR: progesteron receptor

*p-value according to Kruskal-Wallis, Chi square test or Fisher’s exact test, as appropriate

Local treatments (surgery, axillary nodal assessment and radiotherapy) are reported in Table 2.

**Table 2:** Local-regional treatments.

Local-regional treatment	All patients, n = 303	No AST, n = 34	AST without trastuzumab, n = 65	AST with trastuzumab, n = 204
**Surgery**				
Conservative	232 (77%)	16 (47%)	57 (88%)	159 (78%)
Mastectomy	71 (23%)	18 (53%)	8 (12%)	45 (22%)
**Axillary dissection**				
No	253 (83%)	26 (76%)	57 (88%)	179 (83%)
Yes	45 (15%)	8 (24%)	8 (12%)	29 (14%)
Unknown	5 (2%)	0 (0%)	0 (0%)	5 (2%)
**Radiotherapy**				
No	104 (34%)	23 (68%)	22 (34%	59 (29%)
Yes	199 (66%)	11 (32%)	43 (66%)	145 (71%)

AST: adjuvant systemic therapy

The adjuvant systemic therapies are summarized in Table 3. Only 34 (11%) patients did not receive any adjuvant systemic treatment. Most of them were post-menopausal (26/34, 76%), had a pT1a tumor (29/34, 85%) and had negative hormonal receptor status (30/34, 88%). Adjuvant systemic therapy without trastuzumab was given to 65 patients. Among them, 61 (94%) had positive hormonal receptor status, and 58 (89%) received endocrine therapy only. The majority of patients (204/303, 66%) received adjuvant systemic therapy with trastuzumab. In this group, adjuvant systemic therapy was chemotherapy with or without endocrine therapy in 93% of cases, whereas only 7% of patients received endocrine therapy with trastuzumab.

**Table 3:** Adjuvant systemic therapies.

Type of AST	All patients, n = 303	No AST, n = 34	AST without trastuzumab, n = 65	AST with trastuzumab, n = 204
**No systemic therapy**	34 (11%)	34 (100%)	-	-
**CT**	79 (26%)	-	5 (8%)	74 (36%)
**HT**	71 (23%)	-	58 (89%)	13 (6%)
**CT + HT**	119 (39%)	-	2 (3%)	117 (57%)

AST: adjuvant systemic therapy; CT: chemotherapy; HT: hormone therapy

### Univariate analysis

Table 1 shows the univariate analysis of the association between clinicopathological characteristics and the three different systemic adjuvant strategies: no adjuvant systemic therapy, adjuvant systemic therapy without trastuzumab and adjuvant systemic therapy with trastuzumab.

The distribution of age, menopausal status, histology, tumor size, tumor grading, Ki67 and hormone receptors status were significantly heterogeneous among the three treatment groups. In particular, patients treated with adjuvant systemic therapy plus trastuzumab had more frequently pT1b tumors than patients who did not receive any adjuvant systemic therapy (76% vs 15%).

Median age in the adjuvant systemic therapy with trastuzumab group was lower than the other two groups of treatment (55 years for adjuvant systemic therapy with trastuzumab vs 59 years for both the other two groups). Consistently with this finding, patients in the adjuvant systemic therapy with trastuzumab group were also more often pre-menopausal (36%) when compared with those in the adjuvant systemic therapy without trastuzumab group (23%) and in the no adjuvant systemic therapy group (21%). Tumors of patients receiving adjuvant systemic therapy with trastuzumab had more aggressive biologic features such as high proliferation index and high grade, when compared with patients receiving adjuvant systemic therapy without trastuzumab (respectively, Ki67 ≥ 14%: 79% vs 58%; G3: 52% vs 24%).

Hormone receptor status was positive in 12%, 66% and 94% in the no adjuvant systemic therapy group, adjuvant systemic therapy with trastuzumab group and adjuvant systemic therapy without trastuzumab group, respectively. The high percentage of patients in the adjuvant systemic therapy without trastuzumab group treated with endocrine therapy only (58/65; 83%) is possibly related to the high prevalence of hormone positive tumors (94%) in this group (Tables 1 and 3).

### Multivariate analysis

Considering the group of patients treated with adjuvant systemic therapy with trastuzumab as a reference group, multivariate analysis (Table 4) shows the odd ratios of not being treated or of receiving adjuvant systemic therapy alone according to the clinicopathological characteristics. In the multivariate analysis, age, tumor size, Ki67 and hormone receptor status were significantly associated with a different probability for patients of being included in one treatment group or in one other. Patients with older age had more chance of receiving no adjuvant systemic therapy or adjuvant systemic therapy without trastuzumab than with trastuzumab (odds ratio 1.10, 95% CI 1.03–1.18, and 1.03, 95% CI 0.99–1.07, respectively; p = 0.007). Patients with pT1b tumors less frequently did not receive adjuvant systemic therapy or received adjuvant systemic therapy without trastuzumab than adjuvant systemic therapy with trastuzumab (odds ratio 0.06, 95% CI 0.02–0.20, and 0.35, 95% CI 0.17–0.72, respectively; p<.001). Similarly, patients whose tumors had a high proliferation index had less chance to not receive adjuvant systemic therapy or to be treated with adjuvant systemic therapy without trastuzumab than to receive adjuvant systemic therapy with trastuzumab (odds ratio 0.24, 95% CI 0.07–0.79, and 0.53, 0.27–1.05, respectively; p = 0.019). Patients with positive hormone receptor status were less frequently included in the no adjuvant systemic therapy than in the adjuvant systemic therapy with trastuzumab group (odd ratio 0.06, 95% CI 0.01–0.24), but they had more chance of receiving adjuvant systemic therapy without trastuzumab than with trastuzumab (odd ratio 8.87, 95% CI 2.55–30.84; p<0.001).

**Table 4:** Multivariate analysis.

Variable	Systemic therapy	Odds ratio (95% CI)	p value
**Age (**as continuous variable)	No AST *vs* AST + trastuzumab	1.10 (1.03–1.18)	0.007
	AST *vs* AST + trastuzumab	1.03 (0.99–1.07)	
**Menopausal status** (Post vs Pre)	No AST *vs* AST + trastuzumab	1.28 (0.27–6.16)	0.875
	AST *vs* AST + trastuzumab	1.24 (0.47–3.27)	
**Stage** (pT1b vs pT1a)	No AST *vs* AST + trastuzumab	0.06 (0.02–0.20)	<.001
	AST *vs* AST + trastuzumab	0.35 (0.17–0.72)	
**Histology** (Others vs Ductal)	No AST *vs* AST + trastuzumab	7.90 (0.90–69.12)	0.168
	AST *vs* AST + trastuzumab	0.99 (0.21–4.50)	
**Grading** (G3 vs G1)	No AST *vs* AST + trastuzumab	0.81 (0.06–10.11)	0.221
	AST *vs* AST + trastuzumab	0.45 (0.11–1.82)	
**HR status** (Positive vs Negative)	No AST *vs* AST + trastuzumab	0.06 (0.01–0.24)	<.001
	AST *vs* AST + trastuzumab	8.87 (2.55–30.84)	
**Ki67** (≥ 14% vs <14%)	No AST *vs* AST + trastuzumab	0.24 (0.07–0.79)	0.019
	AST *vs* AST + trastuzumab	0.53 (0.27–1.05)	

AST: adjuvant systemic therapy; HR: hormone receptors

### Treatment efficacy

**Fig 1:**
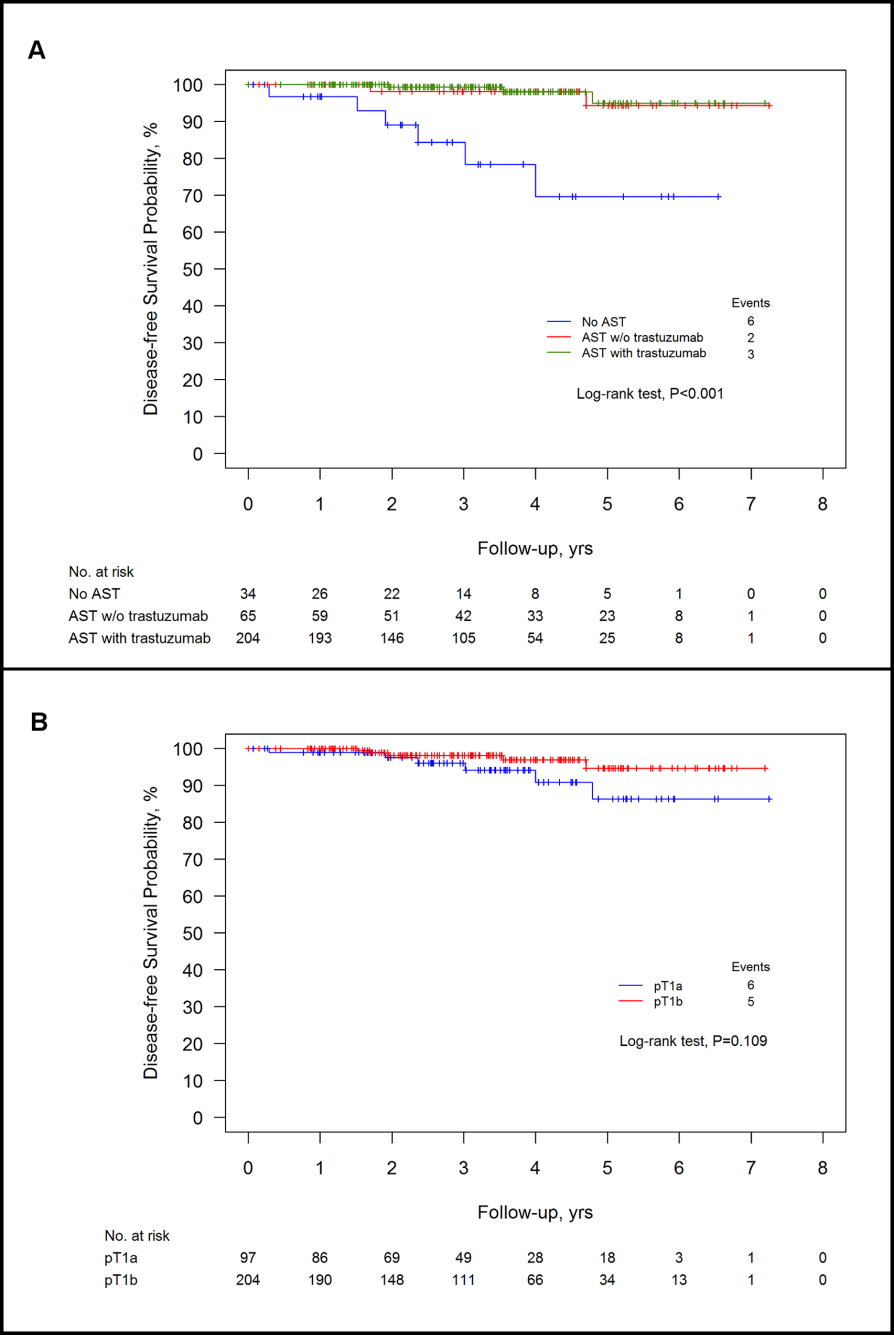
A. Disease-free survival according to treatment group. Fig 1A shows Kaplan Meier’s disease-free survival curves according to treatment group. B. Disease-free survival according to stage. Fig 1B shows Kaplan Meier’s disease-free survival curves according to stage; the analysis was performed on 301 patients, since data on stage were missing for 2 patients.

After 38.6 months of median follow-up (IQR 23.4–53.9 months) local and/or distant recurrences were 5/34 (15%) in the no adjuvant systemic therapy group, 2/65 (3%) in the adjuvant systemic therapy without trastuzumab group, and 3/204 (1.5%) in the adjuvant systemic therapy with trastuzumab group. A statistically significant difference (p<0.001) in 5-years disease-free survival was observed among groups: 69.6% in the no adjuvant systemic therapy group, 94.3% in the adjuvant systemic therapy without trastuzumab group and 95% in the adjuvant systemic therapy with trastuzumab group (no adjuvant systemic therapy vs adjuvant systemic therapy without trastuzumab, adjusted p = 0.007; no adjuvant systemic therapy vs adjuvant systemic therapy with trastuzumab, adjusted p<0.001; adjuvant systemic therapy without trastuzumab versus adjuvant systemic therapy with trastuzumab, adjusted p = 0.621). Kaplan Meier’s disease-free survival curves according to treatment group and stage are shown in Fig 1A and 1B. We observed one death without recurrence in the no adjuvant systemic therapy group.

The characteristics of patients who developed recurrent disease are reported in Table 5.

**Table 5:** Characteristics of patients with relapse.

Systemic Therapy	Patient n.	Age	Menopausal status	Histology	Tumor Size	Grading	LVI	ER status	PgR status	Ki67 (%)	Surgery	Radiotherapy	Pattern of relapse	RFS (months)
**No AST**	#1	35	Pre	Ductal	pT1a	G2	NA	Negative	Negative	20–30	Mastectomy	No	Local and distant	48.1
	#2	76	Post	Other	pT1b	G3	No	Negative	Negative	14–19	Mastectomy	No	Distant	18.3
	#3	58	Post	Ductal	pT1a	G3	No	Negative	Negative	20–30	Mastectomy	No	Local	28.4
	#4	66	Post	Ductal	pT1a	G3	No	Negative	Negative	<14	Conservative	Yes	Local and distant	36.3
	#5	57	Post	Ductal	pT1a	G2	No	Negative	Negative	<14	Conservative	No	Local	3.5
**AST without trastuzumab**	#6	53	Post	Ductal	pT1b	G2	No	Positive	Negative	≥31	Conservative	Yes	Local and distant	56.4
	#7	39	Pre	Ductal	pT1b	G2	No	Positive	Positive	14–19	Conservative	No	Distant	20.4
**AST with trastuzumab**	#8	42	Post	Ductal	pT1b	G2	Yes	Negative	Negative	≥31	Mastectomy	No	Distant	NA
	#9	61	Post	Ductal	pT1a	G3	No	Negative	Negative	NA	Conservative	Yes	Distant	57.5
	#10	43	Pre	Ductal	pT1b	G3	No	Positive	Positive	14–19	Mastectomy	No	Local	23.4

LVI: lymphovascular invasion; ER: estrogen receptor; PgR: progesteron receptor; RFS: relapse-free survival; AST: adjuvant systemic therapy; NA: not available

## Discussion

In our retrospective study, 269 out of 303 (89%) patients undergoing surgery from 2007 to 2012 for pT1a-b pN0 breast cancer in 28 Italian sites received adjuvant systemic therapy, and 204 patients (66%) received adjuvant systemic therapy with trastuzumab. The majority of patients treated with adjuvant systemic therapy plus trastuzumab (191/204; 93%) received chemotherapy.

The high number of patients treated with chemotherapy plus trastuzumab suggests that medical oncologists in Italy feel that HER2-positive tumors have a more aggressive behavior, and that adjuvant trastuzumab may be beneficial in most cases.

This study aimed to identify the clinical and pathological characteristics that may have influenced oncologists in the choice of adjuvant systemic therapeutic strategy. We observed a different distribution among the three different groups of treatment (no adjuvant systemic therapy, adjuvant systemic therapy with trastuzumab and without trastuzumab) of the following characteristics that may have conditioned the treatment choice: age, menopausal status, histology, tumor size, grading, Ki67 and hormone receptor status. In the multivariate analysis tumor size, Ki67, age and hormone receptor status were significantly associated with the administration of adjuvant systemic therapy with trastuzumab (Table 4).

Tumor size seems to have played an important role in the choice of treatment, as shown by multivariate analysis (p = <.001) (Table 4). In fact, the patients who did not receive any adjuvant systemic therapy had mainly pT1a tumors (29/34, 85%; Table 1), whereas patients treated with adjuvant systemic therapy plus trastuzumab had more frequently pT1b tumors (156/204, 76%; Table 1). Tumor size is a relevant prognostic factor for early breast cancer patients and data from US National Cancer Database showed that among 123,212 patients with pT1a-b pN0 breast cancer, unselected for HER2 status, 5-year survival rates were marginally but significantly higher for pT1a than pT1b (94.3% vs 93,1%, p = 0.04) [25]. In fact, in our study we observed that patients with pT1b tumors were treated with adjuvant systemic therapy plus trastuzumab more frequently than those with pT1a tumors (156/204, 76% vs 46/97, 47%; Table 1).

On the other hand, the tumor size alone was not sufficient for the Italian oncologists to establish the type of adjuvant treatment. In fact, 47% of pT1a pN0 HER2-positive breast cancer patients (46 out of 97 patients with pT1a tumors) received adjuvant systemic therapy with trastuzumab, because other bio-pathological and clinical characteristics had an impact on the treatment choice, such as proliferation index, age, and hormone receptor status.

The proliferation index was another parameter significantly associated with the choice of therapy in our study (p = 0.015 at the multivariate analysis; Table 4) and adjuvant systemic therapy plus trastuzumab was administered more frequently to patients with high Ki67. The prognostic role of Ki67 was shown in a meta-analysis of 40 studies on 12.000 patients, where high levels of Ki67 were associated with a worse survival [26]

Patients treated with adjuvant systemic therapy plus trastuzumab were younger than patients who received adjuvant systemic therapy without trastuzumab or no systemic therapy, with statistical significance at multivariate analysis (p = 0.007).Probably this was due to the fact that increasing age is often associated with cardiovascular comorbidities which enhance the risk for treatment-related adverse events, especially in unselected elderly patients treated outside of clinical trials. In a retrospective population-based study, most elderly patients completed the therapy with adjuvant trastuzumab, but 3.6% of patients were hospitalized for cardiotoxicity [27]. We have no sufficient data about safety of adjuvant trastuzumab in the elderly due to the limited number of older pts enrolled in clinical trials. Median age of patients enrolled in the HERA trial was 49 years [14], and there are not many data about safety of adjuvant trastuzumab in the elderly due to the limited number of older patients enrolled in clinical trials. In the HERA trial, at a median follow-up of 3.6 years, among 1,703 patients treated with trastuzumab for 1 year, severe heart failure and significant decrease of the ejection fraction were reported in 0.8% and 3.6% of patients respectively, compared with 0% and 0.6% reported in the observation arm of the study [28]. It is well-known that increasing age is often associated with cardiovascular comorbidities which enhance the risk for treatment-related cardiac events, especially in unselected elderly patients treated outside from clinical trials. In a retrospective population-based study, most elderly patients completed the therapy with adjuvant trastuzumab, but 3.6% of patients were hospitalized for cardiac toxicity [28]. Age and comorbidities were factor associated with treatment completion and with incidence of cardiac adverse events. We did not collect data about comorbidities in detail, therefore we could not say how comorbidities influenced the choice for the adjuvant treatment.

In our study, hormone receptor status significantly affected the decision about the adjuvant therapeutic strategy. The distribution of hormone receptor positive and negative tumors in the adjuvant systemic therapy plus trastuzumab group (n = 204) was similar to all patients (Table 1), while the main difference in terms of distribution of hormone receptor status was observed between the no adjuvant systemic therapy group (n = 34) where there was a prevalence of hormone receptor negative tumors (88%) and the adjuvant systemic therapy without trastuzumab group (n = 65) where there was a prevalence of hormone receptor positive tumors (94%) and 89% of patients received only endocrine therapy. In the multivariate analysis, patients with hormone receptor positive tumors received more frequently adjuvant systemic therapy alone than adjuvant systemic therapy plus trastuzumab (odds ratio 8.87, 95% IC 2.55–30.84; p<0.001). Since among HER2-positive disease, hormone receptor positive tumors have a better prognosis than hormone receptor negative tumors [29], this may explain the decision of oncologists to avoid trastuzumab and prescribe adjuvant endocrine therapy alone in a subgroup of patients with hormone receptor positive, HER2-positive, node-negative small breast cancer.

After a median follow-up of 38.6 months, the recurrence rate was higher for patients not treated with adjuvant systemic therapy (15%) compared with those receiving adjuvant systemic therapy plus trastuzumab (1.5%). This finding adds to a growing body of evidence on the potential benefit of adjuvant trastuzumab in HER2-positive small tumors with negative nodes [21–23]. Interestingly, 5-year disease-free survival probabilty rates were 95% for adjuvant systemic therapy with trastuzumab group and 94.3% for adjuvant systemic therapy without trastuzumab group (adjusted p = 0.621). Since in the adjuvant systemic therapy without trastuzumab group were included mainly patients with hormone receptor positive tumors receiving endocrine adjuvant therapy, these results suggest that trastuzumab could be avoided in selected cases of hormone positive breast cancer.

However, no definitive conclusion may be drawn about the efficacy of trastuzumab in this setting, due to many limitations of our study. First, our study is a retrospective study with a limited sample size, not specifically designed to detect a difference of outcomes between patients receiving trastuzumab and those not receiving trastuzumab. Second, the follow-up time is relatively short.Our data suggest that adjuvant systemic therapy could be beneficial also for pT1a pN0 tumors. Even if pT1a has generally a better prognosis than pT1b tumors, Houvenaeghel et al reported a worse relapse-free survival for pT1a compared with pT1b tumors [30]. One of the hypotheses reported by the authors is that the lack of adjuvant trastuzumab-based treatment in pT1a HER2-positive tumors could have contributed to a less favorable outcome.

Although the pT1a-b pN0 breast cancer generally has a good prognosis, it is now recognized that molecular subtypes affects the outcome. In particular, triple-negative and HER2-positive tumors have a worse prognosis than hormone receptor positive tumors, even for node negative and ≤ 1cm in size breast carcinoma [5–8,10].

Population with pT1a-b pN0 breast cancer was not represented in phase III randomized trials of adjuvant trastuzumab [11–19], since most of the studies included only node positive or high risk node negative breast cancers, with primary tumors size ≥ 2 cm.

Only the BCIRG 006 trial, which randomized into 3 arms (AC→T vs AC→TH vs TCH arm) 3,222 women with HER2 positive breast cancer and included patients with node positive or high risk node negative disease (i.e. with at least one of the following risk factors: tumor size > 2 cm, histologic and/or nuclear grade 2–3, age < 35, hormone receptor negative status), enrolled 148 women with tumor size <1 cm [16]. The 5-year disease-free survival rate for patients with <1 cm tumours was 86% for both trastuzumab-containing arms compared with 72% for chemotherapy alone. However, the nodal status of patients with these small tumors was not reported by the authors, therefore no definitive conclusions can be drawn on the efficacy of trastuzumab in the adjuvant therapy for HER2 positive pT1a-b pN0 breast cancer.

Recently, in a large single-arm prospective clinical trial, 406 patients with HER2-positive, node negative breast cancer up to 3 cm were treated with adjuvant paclitaxel and trastuzumab, with a 3-year rate of survival free from invasive disease of 98.7% [20]. The study enrolled 192 patients with pT1a-b pN0 breast cancer, and the outcome of patients with ≤ 1 cm tumors did not differ from that of patients with larger tumors, underlying the role of adjuvant trastuzumab in this setting. Similarly, in a single-arm phase 2 study on 493 patients with early stage HER2 positive breast cancer treated with a four-cycle regimen of docetaxel and cyclophosphamide combined with trastuzumab, 95 patients with node negative, ≤ 1 cm tumors had 100% of 3-year disease-free and overall survival rate. These data suggest that a depotentiated and possibly less toxic regimen could be an option for patients with HER2-positive small tumors.

## Conclusion

From 2007 to 2012, in Italian daily clinical practice, 66% of patients with HER2-positive, pT1a-b pN0 breast cancer received adjuvant systemic therapy plus trastuzumab. The main clinical and pathological characteristics that influenced physicians in the choice of treatment were tumor size, proliferation index, hormone receptor status and the age of patients. After 38.6 months of median follow-up, the highest recurrence rate was observed in the no adjuvant systemic therapy group (15%), with a small difference in 5-year disease-free survival between adjuvant systemic therapy with trastuzumab and adjuvant systemic therapy without trastuzumab group (95% and 94.3%, respectively). It should be emphasized, however, that in the adjuvant systemic therapy with trastuzumab group 94% of tumors were hormone receptor positive and 89% of patients were treated with endocrine therapy only. Our study adds further data to the growing evidence that adjuvant trastuzumab treatment is beneficial to the prognosis of HER2-positive pT1a-b pN0 breast cancer, but selected patients with hormone receptor positive tumors might benefit from endocrine therapy only.

## References

[1] Edge S, Byrd DR, Compton CC, Fritz AG, Greene FL, Trotti A (2010) AJCC Cancer Staging Manual (ed. 7). Springer-Verlag New York

[2] Schmidt F, Hartwagner KA, Spork EB, Groell R (1998) Medical audit after 26,711 breast imaging studies: improved rate of detection of small breast carcinomas (classified as Tis or T1a,b). Cancer 83:2516–2520. 9874457 10.1002/(sici)1097-0142(19981215)83:12<2516::aid-cncr16>3.0.co;2-#

[3] Hanrahan EO, Gonzalez-Angulo AM, Giordano SH, Rouzier R, Broglio KR, Hortobagyi GN, et al (2007) Overall survival and cause-specific mortality of patients with stage T1a,bN0M0 breast carcinoma. J Clin Oncol 25:4952–4960. 17971593 10.1200/JCO.2006.08.0499

[4] Slamon DJ1, Godolphin W, Jones LA, Holt JA, Wong SG, Keith DE, et al (1989) Studies of the HER-2/neu proto-oncogene in human breast and ovarian cancer. Science 244:707–712. 2470152 10.1126/science.2470152

[5] Curigliano G, Viale G, Bagnardi V, Fumagalli L, Locatelli M, Rotmensz N, et al (2009) Clinical relevance of HER2 overexpression/amplification in patients with small tumor size and node-negative breast cancer. J Clin Oncol 27:5693–5699. 10.1200/JCO.2009.22.0962 19884553

[6] Gonzalez-Angulo AM, Litton JK, Broglio KR, Meric-Bernstam F, Rakkhit R, Cardoso F, et al (2009) High risk of recurrence for patients with breast cancer who have human epidermal growth factor receptor 2-positive, node-negative tumors 1 cm or smaller. J Clin Oncol 27:5700–5706 10.1200/JCO.2009.23.2025 19884543 PMC2792998

[7] Joensuu H, Isola J, Lundin M, Salminen T, Holli K, Kataja V, et al (2003) Amplification of erbB2 and erbB2 expression are superior to estrogen receptor status as risk factors for distant recurrence in pT1N0M0 breast cancer: a nationwide population-based study. Clin Cancer Res 9:923–930. 12631589

[8] Petrelli F, Barni S (2012) Role of HER2-neu as a prognostic factor for survival and relapse in pT1a-bN0M0 breast cancer: a systematic review of the literature with a pooled-analysis. Med Oncol 29:2586–2593. 10.1007/s12032-012-0201-4 22415399

[9] Fehrenbacher L, Capra AM, Quesenberry CP Jr, Fulton R, Shiraz P, Habel LA (2014) Distant invasive breast cancer recurrence risk in human epidermal growth factor receptor 2-positive T1a and T1b node-negative localized breast cancer diagnosed from 2000 to 2006: a cohort from an integrated health care delivery system. J Clin Oncol 32:2151–2158. 10.1200/JCO.2013.52.0858 24888815

[10] Vaz-Luis I, Ottesen RA, Hughes ME, Mamet R, Burstein HJ, Edge SB, et al (2014) Outcomes by tumor subtype and treatment pattern in women with small, node-negative breast cancer: a multi-institutional study. J Clin Oncol 32:2142–2150. 10.1200/JCO.2013.53.1608 24888816 PMC4076026

[11] Romond EH, Perez EA, Bryant J, Suman VJ, Geyer CE Jr, Davidson NE, et al (2005) Trastuzumab plus adjuvant chemotherapy for operable HER2-positive breast cancer. N Engl J Med 353:1673–1684. 16236738 10.1056/NEJMoa052122

[12] Perez EA, Romond EH, Suman VJ, Jeong JH, Sledge G, Geyer CE Jr, et al (2014) Trastuzumab plus adjuvant chemotherapy for human epidermal growth factor receptor 2-positive breast cancer: planned joint analysis of overall survival from NSABP B-31 and NCCTG N9831. J Clin Oncol 32:3744–3752. 10.1200/JCO.2014.55.5730 25332249 PMC4226805

[13] Perez EA, Suman VJ, Davidson NE, Gralow JR, Kaufman PA, Visscher DW, et al (2011) Sequential versus concurrent trastuzumab in adjuvant chemotherapy for breast cancer. J Clin Oncol 29:4491–4497. 10.1200/JCO.2011.36.7045 22042958 PMC3236650

[14] Piccart-Gebhart MJ, Procter M, Leyland-Jones B, Goldhirsch A, Untch M, Smith I, et al (2005) Trastuzumab after adjuvant chemotherapy in HER2-positive breast cancer. N Engl J Med 353:1659–1672. 16236737 10.1056/NEJMoa052306

[15] Gianni L, Dafni U, Gelber RD, Azambuja E, Muehlbauer S, Goldhirsch A, et al (2011) Treatment with trastuzumab for 1 year after adjuvant chemotherapy in patients with HER2-positive early breast cancer: a 4-year follow-up of a randomised controlled trial. Lancet Oncol 12:236–244. 10.1016/S1470-2045(11)70033-X 21354370

[16] Slamon D, Eiermann W, Robert N, Pienkowski T, Martin M, Press M, et al (2011) Adjuvant trastuzumab in HER2-positive breast cancer. N Engl J Med 365:1273–1283. 10.1056/NEJMoa0910383 21991949 PMC3268553

[17] Joensuu H, Kellokumpu-Lehtinen PL, Bono P, Alanko T, Kataja V, Asola R, et al (2006) Adjuvant docetaxel or vinorelbine with or without trastuzumab for breast cancer N Engl J Med 354:809–820. 16495393 10.1056/NEJMoa053028

[18] Joensuu H, Bono P, Kataja V, Alanko T, Kokko R, Asola R, et al (2009) Fluorouracil, epirubicin, and cyclophosphamide with either docetaxel or vinorelbine, with or without trastuzumab, as adjuvant treatments of breast cancer: final results of the FinHer Trial. J Clin Oncol 27:5685–5692. 10.1200/JCO.2008.21.4577 19884557

[19] Spielmann M, Roché H, Delozier T, Canon JL, Romieu G, Bourgeois H, et al (2009) Trastuzumab for patients with axillary-node-positive breast cancer: results of the FNCLCC-PACS 04 trial. J Clin Oncol 27:6129–6134. 10.1200/JCO.2009.23.0946 19917839

[20] Tolaney SM, Barry WT, Dang CT, Yardley DA, Moy B, Marcom PK, et al (2015) Adjuvant paclitaxel and trastuzumab for node-negative, HER2-positive breast cancer. N Engl J Med 372:134–141. 10.1056/NEJMoa1406281 25564897 PMC4313867

[21] McArthur HL, Mahoney KM, Morris PG, Patil S, Jacks LM, Howard J, et al (2011) Adjuvant trastuzumab with chemotherapy is effective in women with small, node-negative, HER2-positive breast cancer. Cancer 117:5461–5468. 10.1002/cncr.26171 21681735

[22] Rodrigues MJ, Peron J, Frénel JS, Vano YA, Wassermann J, Debled M, et al (2013) Benefit of adjuvant trastuzumab-based chemotherapy in T1ab node-negative HER2-overexpressing breast carcinomas: a multicenter retrospective series. Ann Oncol 24:916–924. 10.1093/annonc/mds536 23104720

[23] Horio A, Fujita T, Hayashi H, Hattori M, Kondou N, Yamada M, et al (2012) High recurrence risk and use of adjuvant trastuzumab in patients with small, HER2-positive, node-negative breast cancers. Int J Clin Oncol 17:131–136. 10.1007/s10147-011-0269-4 21681642

[24] Zhou Q, Yin W, Du Y, Lu J (2014) For or against adjuvant trastuzumab for pT1a-bN0M0 breast cancer patients with HER2-positive tumors: a meta-analysis of published literatures. PLoS One 9:e83646 10.1371/journal.pone.0083646 24392090 PMC3879252

[25] Kennedy T, Stewart AK, Bilimoria KY, Patel-Parekh L, Sener SF, Winchester DP (2007) Treatment trends and factors associated with survival in T1aN0 and T1bN0 breast cancer patients. Ann Surg Oncol 14:2918–2927. 17638060 10.1245/s10434-007-9441-5

[26] de Azambuja E, Cardoso F, de Castro G Jr, Colozza M, Mano MS, Durbecq V, et al (2007) Ki-67 as prognostic marker in early breast cancer: a meta-analysis of published studies involving 12,155 patients. Br J Cancer 96:1504–1513. 17453008 10.1038/sj.bjc.6603756 PMC2359936

[27] Vaz-Luis I, Keating NL, Lin NU, Lii H, Winer EP, Freedman RA (2014) Duration and toxicity of adjuvant trastuzumab in older patients with early-stage breast cancer: a population-based study. J Clin Oncol 32:927–934. 10.1200/JCO.2013.51.1261 24516021 PMC3948095

[28] Procter M, Suter TM, de Azambuja E, Dafni U, van Dooren V, Muehlbauer S, et al (2010) Longer-term assessment of trastuzumab-related cardiac adverse events in the Herceptin Adjuvant (HERA) trial. J Clin Oncol 28:3422–3428. 10.1200/JCO.2009.26.0463 20530280

[29] Kolben T, Harbeck N, Wuerstlein R, Schubert-Fritschle G, Bauerfeind I, Schrodi S, et al (2015) Endocrine sensitivity is decisive for patient outcome in small node-negative breast cancers (BC) (pT1a,b)—Results from the Munich Cancer Registry. Breast 24:24–31. 10.1016/j.breast.2014.10.007 25543874

[30] Houvenaeghel G, Goncalves A, Classe JM, Garbay JR, Giard S, Charytensky H, et al (2014) Characteristics and clinical outcome of T1 breast cancer: a multicenter retrospective cohort study. Ann Oncol 25:623–628. 10.1093/annonc/mdt532 24399079 PMC4433506

