# Correction: The Promher Study: An Observational Italian Study on Adjuvant Therapy for HER2-Positive, pT1a-b pN0 Breast Cancer

**DOI:** 10.1371/journal.pone.0139650

**Published:** 2015-09-25

**Authors:** Stefania Gori, Alessandro Inno, Elena Fiorio, Jennifer Foglietta, Antonella Ferro, Marcella Gulisano, Graziella Pinotti, Marta Gubiotti, Maria Giovanna Cavazzini, Monica Turazza, Simona Duranti, Valeria De Simone, Laura Iezzi, Giancarlo Bisagni, Simon Spazzapan, Luigi Cavanna, Chiara Saggia, Emilio Bria, Elisabetta Cretella, Patrizia Vici, Daniele Santini, Alessandra Fabi, Ornella Garrone, Antonio Frassoldati, Laura Amaducci, Silvana Saracchini, Lucia Evangelisti, Sandro Barni, Teresa Gamucci, Lucia Mentuccia, Lucio Laudadio, Alessandra Zoboli, Fabiana Marchetti, Giuseppe Bogina, Gianluigi Lunardi, Luca Boni

There is an error in the second to last sentence of the Conclusion. The correct sentence is: It should be emphasized, however, that in the adjuvant systemic therapy without trastuzumab group 94% of tumors were hormone receptor positive and 89% of patients were treated with endocrine therapy only.
